# Unique Structure and Stability of HmuY, a Novel Heme-Binding Protein of *Porphyromonas gingivalis*

**DOI:** 10.1371/journal.ppat.1000419

**Published:** 2009-05-08

**Authors:** Halina Wójtowicz, Tibisay Guevara, Cynthia Tallant, Mariusz Olczak, Aneta Sroka, Jan Potempa, Maria Solà, Teresa Olczak, F. Xavier Gomis-Rüth

**Affiliations:** 1 Laboratory of Biochemistry, Faculty of Biotechnology, University of Wroclaw, Wroclaw, Poland; 2 Proteolysis Lab, Department of Structural Biology, Molecular Biology Institute of Barcelona, CSIC, Barcelona, Spain; 3 Laboratory of Microbiology, Faculty of Biochemistry, Biophysics and Biotechnology, Jagiellonian University, Krakow, Poland; 4 Department of Periodontics, University of Louisville School of Dentistry, Louisville, Kentucky, United States of America; 5 Structural MitoLab, Department of Structural Biology, Molecular Biology Institute of Barcelona, CSIC, Barcelona, Spain; The Rockefeller University, United States of America

## Abstract

Infection, survival, and proliferation of pathogenic bacteria in humans depend on their capacity to impair host responses and acquire nutrients in a hostile environment. Among such nutrients is heme, a co-factor for oxygen storage, electron transport, photosynthesis, and redox biochemistry, which is indispensable for life. *Porphyromonas gingivalis* is the major human bacterial pathogen responsible for severe periodontitis. It recruits heme through HmuY, which sequesters heme from host carriers and delivers it to its cognate outer-membrane transporter, the TonB-dependent receptor HmuR. Here we report that heme binding does not significantly affect the secondary structure of HmuY. The crystal structure of heme-bound HmuY reveals a new all-β fold mimicking a right hand. The thumb and fingers pinch heme iron through two apical histidine residues, giving rise to highly symmetric octahedral iron co-ordination. The tetrameric quaternary arrangement of the protein found in the crystal structure is consistent with experiments in solution. It shows that thumbs and fingertips, and, by extension, the bound heme groups, are shielded from competing heme-binding proteins from the host. This may also facilitate heme transport to HmuR for internalization. HmuY, both in its apo- and in its heme-bound forms, is resistant to proteolytic digestion by trypsin and the major secreted proteases of *P. gingivalis*, gingipains K and R. It is also stable against thermal and chemical denaturation. In conclusion, these studies reveal novel molecular properties of HmuY that are consistent with its role as a putative virulence factor during bacterial infection.

## Author Summary

Pathogenic bacteria cause infection in humans as found in periodontitis, which is a chronic inflammation of the gums caused by *Porphyromonas gingivalis*. As part of the infective process, bacteria must acquire nutrients to survive and multiply at the infection site, and among such nutrients is heme. This is an iron-dependent co-factor of several indispensable enzymes and proteins. *P. gingivalis* liberates heme from host heme-binding proteins through the action of proteases and arranges its transport to the bacterial cell through two proteins, HmuY and HmuR. They grab free heme and transport it across the bacterial membrane into the cell, respectively. This function poses stringent conditions on these proteins regarding stability and resistance toward the host immune system. We report here that HmuY is very stable and that it displays a novel protein fold, which consists only of β-strands. It reminds us of a right hand, whose fingers trap heme. Once heme is bound, HmuY forms tetramers, which have the four heme-binding sites buried and thus protected from competing host heme-binding proteins. This feature also facilitates heme transport to HmuR and into the bacterial cell. All these data may help to develop new antibacterial agents at times in which resistance toward antibiotics, both at intensive healthcare stations and in the community, poses serious challenges to human health.

## Introduction

Periodontitis causes chronic inflammation of the gums and it affects 10–15% of adults worldwide, potentially leading to tissue destruction and tooth loss, and *Porphyromonas gingivalis* is its main etiological agent [1],[2]. In addition, *P. gingivalis*, an anaerobic black-pigmented, Gram-negative bacterium, has been implicated in cardiovascular diseases, respiratory diseases, diabetes, osteoporosis, and pre-term low birth-weight [3]-[5]. The pathogen cannot synthesize protoporphyrin IX but acquires exogenous heme (“heme” is here used to refer indistinctly to either Fe^2+^- or Fe^3+^-protoporphyrin IX), an excess of which is stored in the characteristic black pigment on the bacterial cell surface [6]–[8]. The co-factor is obtained from hemoglobin, haptoglobin-hemoglobin, myoglobin, hemopexin, serum albumin, lactoperoxidase, cytochrome c, and catalase by the action of hemolysins and proteases [9]–[12]. In addition, *P. gingivalis* and other Gram-negative bacteria possess systems to bind locally liberated heme such as secreted heme-binding proteins and hemophores [13],[14]. One such hemophore is HasA, employed by *Serratia mercescens* to scavenge host heme in order to deliver it to the receptor, HasR, for internalization [15]. Similarly, hemophores have been described in *Haemophilus influenzae*, *Yersinia enterocolitica*, *Pseudomonas aeruginosa*, and *Bacillus anthracis* [13], [16]–[20]. Further heme is transported into the cell through outer-membrane receptors [21].

In *P. gingivalis*, heme is primarily imported by heme-binding protein, HmuY, and its cognate outer-membrane receptor, HmuR [22]. The latter is involved in heme transport through the outer membrane and probably depends on the interaction with protein TonB, which is needed to transduce energy for the passage of heme and other ligands into the periplasm in most Gram-negative pathogens [11],[14],[22],[23]. The two Hmu proteins are encoded in tandem by the *hmu* operon, which comprises six genes in total, *hmuYRSTUV*. The locus is regulated by iron [23] and by a transcriptional repressor encoded by gene *pg1237* [24], and its disruption leads to a 70% decrease in heme binding and a 45% decrease in heme uptake [25]. Potential protein pairs with high sequence similarity to HmuY and HmuR have been identified on contiguous genes in other bacteroidetes (*Microscilla marina*, *Prevotella intermedia*, and *Bacteroides* from the species *vulgatus*, *fragilis*, *ovatus*, *thetaiotaomicron*, *caccae*, *stercori*, and *coprocola*), proteobacteria (*Plesiocystis pacifica*, *Stigmatella aurantica*, and *Myxococcus xanthus*), spirochaetes (*Leptospira biflexa*), and chlorobi (*Chloroherpeton thalassium*). This suggests a widespread mechanism for heme uptake (our unpublished data; [23],[25]). HmuY is an outer-membrane-associated lipoprotein, which is identical in sequence to a *P. gingivalis* envelope protein designated fibroblast activating factor [26]. This factor induces proliferation and protein synthesis in normal human gingival fibroblasts, indicating an additional role for HmuY in the host immune response. The *hmuY* gene encodes a 23-kDa protein, with no significant sequence similarity to any other protein, whose 25 first residues are not present in the purified protein. This stretch comprises a leader sequence, a lipid-binding site, and a potential protease cleavage site [23],[25]. The protein is functional as a dimer in its heme-depleted form and as a tetramer once heme is bound [23]. Heme bound to HmuY, with a midpoint potential of 136 mV, displays a low-spin six-fold Fe^3+^ co-ordination sphere with the participation of residues His134 and His166, as revealed by point mutation studies [27].

In order to shed light on the mechanisms of heme binding and transport through HmuY, we set out to assess the folding and stability properties of HmuY in its heme-depleted (apo-HmuY) and heme-complexed (holo-HmuY) forms, as well as its susceptibility to proteolysis by trypsin and gingipains K (Kgp) and R (RgpA and RgpB). In addition, we solved the X-ray crystal structure of holo-HmuY. Taken together, these data enabled us to propose a mechanism for Hmu-mediated heme uptake by *P. gingivalis*.

## Results/Discussion

### Conformational stability of HmuY

**Figure 1:**
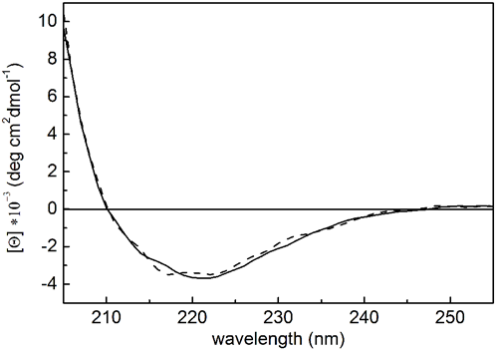
Far-UV CD analysis of P. gingivalis apo- and holo-HmuY. Spectra were recorded for purified HmuY (solid line) and the HmuY-heme complex at a 1∶1 molar ratio (dashed line).

**Figure 2:**
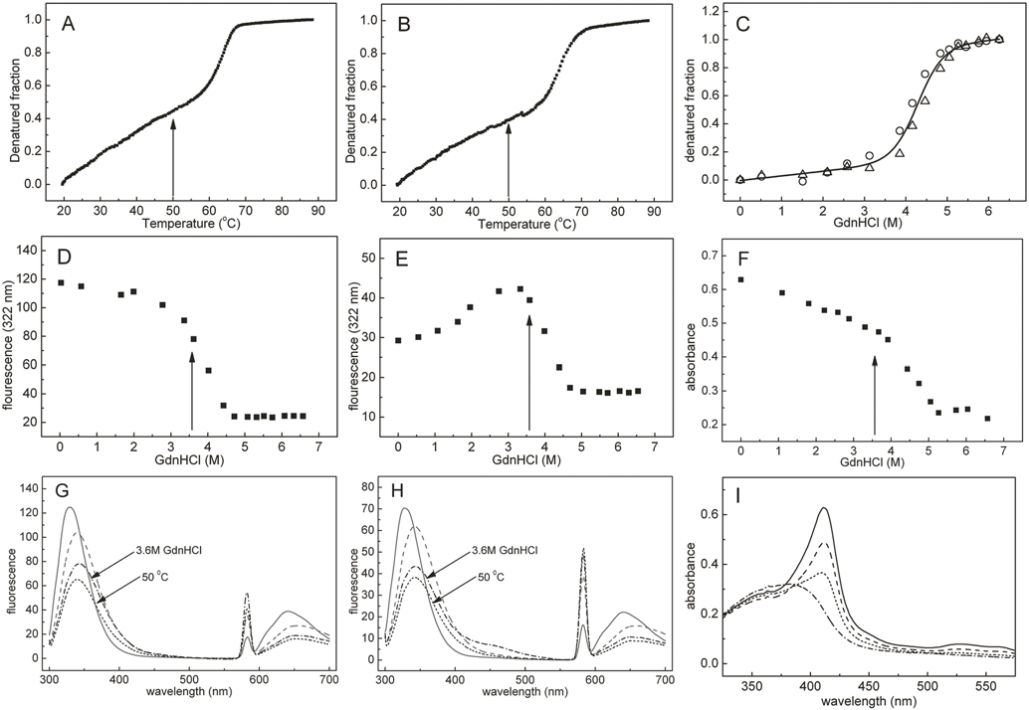
Thermal and chemical unfolding studies. Unfolding transition curves of apo-HmuY (A) and holo-HmuY (B) as a function of temperature measured by tryptophan intrinsic fluorescence. Unfolding transition curves as a function of GdnHCl concentration measured by CD ellipticity at 225 nm (C) for apo-HmuY (circles) and holo-HmuY (triangles). The solid line shows the goodness-of-fit assuming a two-state reverse transition model. Unfolding transition curves as a function of GdnHCl concentration measured by tryptophan intrinsic fluorescence for apo-HmuY (D) and holo-HmuY (E). Heme dissociation from holo-HmuY (F) measured by absorption spectroscopy in the Soret region. Fluorescence spectra of apo-HmuY (G) and holo-HmuY (H): HmuY in 20 mM sodium phosphate, 1 M GdnHCl, pH 6.5 at 20°C before (solid lines), after thermal denaturation and cooling to 20°C (dashed lines), and at 50°C (dotted lines); HmuY in 20 mM sodium phosphate, 20 mM NaCl, 3.6 M GdnHCl, pH 7.4 at 20°C (dashed-dotted lines). (I) Soret region spectra of HmuY-heme: 20 mM sodium phosphate, 20 mM NaCl, pH 7.4 without GdnHCl (solid line), with 3 M (dashed line), 4 M (dotted line), and 6 M (dashed-dotted line) GdnHCl at 20°C.

Assessment of the biophysical response of apo- and holo-HmuY to thermal and chemical denaturation may contribute to unravel heme binding and transport at the infection site. Previously, a far-UV CD spectrum of native apo-HmuY had shown that the protein has mainly a β structure [23]. Here we found that heme binding did not affect the spectrum, indicating absence of significant structural changes of the secondary structure (Figure 1). In unfolding studies (Figure 2), far-UV CD spectroscopy revealed that thermal denaturation of HmuY (encompassing the sequence of the purified natural protein; Asp26-Lys216) was irreversible, leading to protein precipitation (data not shown). In contrast, guanidinium hydrochloride (GdnHCl)-induced chemical denaturation was reversible and no significant difference in the equilibrium unfolding profiles of apo- and holo-HmuY was observed (Figure 2C). Both forms tended to follow one-step unfolding process with a calculated free energy of denaturation (*ΔG_den_*) of 34.1±12.4 kJ/mol. To obtain information on local changes of the tertiary structure of HmuY and the heme cavity, we further studied thermal and chemical denaturation by intrinsic tryptophan fluorescence spectroscopy. Thermal denaturation is reversible and gives rise to sigmoidal unfolding curves for both HmuY forms (Figure 2A and 2B). However, the initial part of the curves, especially that of holo-HmuY, deviates from the one-step reverse-unfolding mechanism. Exposure of apo-HmuY to increasing concentrations of GdnHCl reduced fluorescence intensity, giving a sigmoidal shape to the unfolding curve (Figure 2D). In contrast, the unfolding profile of holo-HmuY (Figure 2E) does not account for a typical one-step reverse-mechanism transition unless the reference wavelength is far away from the heme-binding maximum (323 nm). This suggests that holo-HmuY may exhibit local differences in the tertiary structure when compared with apo-HmuY similar to HasA and HasAp [18],[28],[29]. Simultaneously, we examined heme loss by holo-HmuY by absorbance change in the Soret region (Figure 2F and 2I). Holo-HmuY retained ∼70% of the bound heme up to 3 M GdnHCl, and even in the presence of 4 or 5 M GdnHCl, it remained partially loaded with the co-factor. An interesting feature can be observed at intermediate GdnHCl concentrations (3.6 M) and at ∼50°C. Although the overall fluorescence spectra are similar, thus indicating that the conformations are related (Figure 2G and 2H), the possibility of intermediate species should not be excluded. Changes in the fluorescence spectra in the pre-transitional region may indicate differences in local conformation of HmuY upon heme binding and tetramer formation. This corresponds to the end of the pre-transitional region regardless of the technique used, as indicated by arrows in Figure 2A, 2B, 2D, 2E, and 2F. It may be easier to see the intermediates when heme is bound to HmuY (Figure 2E), since the co-factor modulates the fluorescence characteristics of HmuY tryptophans. We conclude that the first step of the HmuY-heme complex unfolding is a tetramer-to-dimer transition, subsequently leading to heme loss. Posterior dimer dissociation and protein denaturation are probably the limiting steps in this process. Taking all together, HmuY stability against heat and GdnHCl-induced denaturation is similar to or higher than that of other stable proteins bound to heme [30]. However, in contrast to the latter, both apo- and holo-HmuY show comparable resistance to denaturation, although some local changes in the tertiary structure may occur upon heme binding.

### Resistance of HmuY to proteolytic digestion

**Figure 3:**
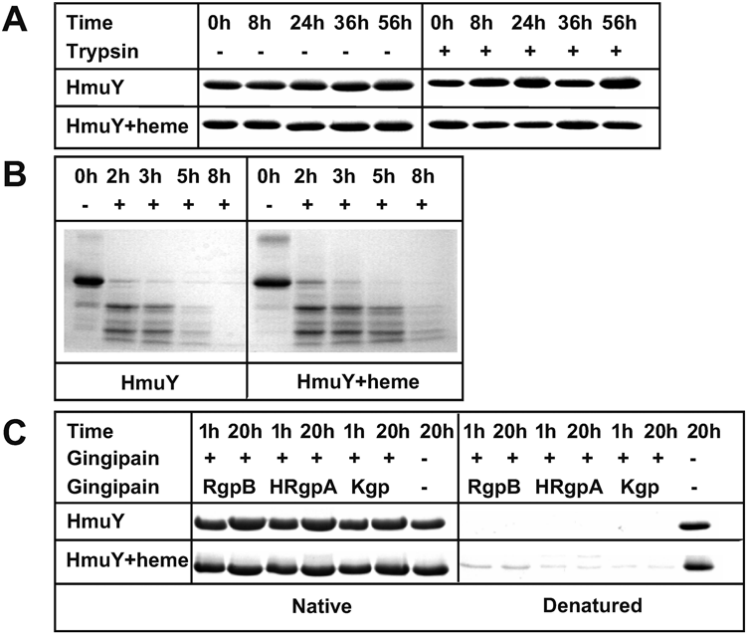
Limited proteolysis experiments. Proteolytic susceptibility of apo- and holo-HmuY (A) in their native states and (B) after thermal denaturation (incubation at 95°C for 10 min) toward trypsin and (C) Kgp, RgpA, and RgpB.

Ligand binding may enhance resistance to proteolysis [31], so trypsin was assayed as a degrading agent against HmuY. In addition, response to the cysteine proteases Kgp, RgpA, and RgpB was examined, as these are secreted by *P. gingivalis* upon infection and target host hemoproteins [12]. Both apo- and holo-HmuY were fully resistant to digestion by trypsin and gingipains in their native state under the conditions assayed (Figure 3). In contrast, protein samples previously subjected to thermal denaturation were completely degraded. These data strongly suggest that HmuY is very stable and compactly folded, regardless of the bound co-factor, and insensitive against endogenous proteases.

### Protomeric structure of holo-HmuY

**Figure 4:**
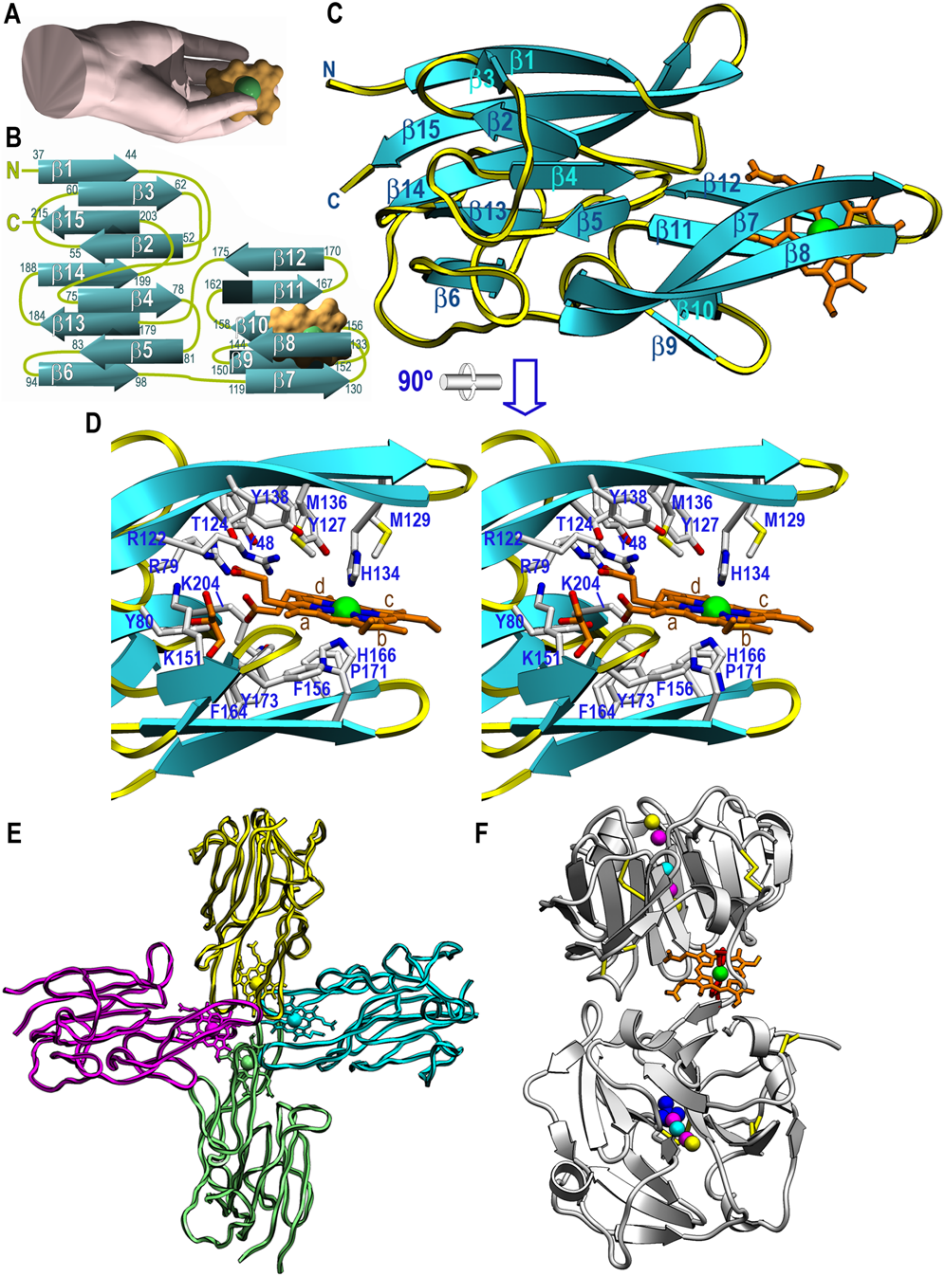
Structure of holo-HmuY. (A) The protein mimics a right hand, whose thumb and fingers trap the heme co-factor (surface model in orange with an inserted green sphere for the iron ion). (B) Topology scheme of HmuY, which consists of 15 β-strands, each labeled with the protein residues it spans. The heme group is shown as in (A). (C) Richardson plot of holo-HmuY Glu35-Lys216 with the bound heme as an orange stick model and an inserted sphere for the iron. The view was chosen to match (A). (D) Close-up view in stereo of (C) after a horizontal 90° rotation. Protein residue side chains engaged in shaping the heme-binding cavity and in interactions with the co-factor are shown and labeled. The four pyrrole rings of the protoporphyrin IX moiety are also labeled (a–d). (E) Tetrameric quaternary arrangement of holo-HmuY. Each constituting heme/HmuY complex is shown in one color. (F) Richardson plot of rabbit serum hemopexin (PDB access code 1QHU; [32]). The two hemopexin-like β-propeller domains contain central channels to bind ions (colored spheres), and the heme-binding site is at the domain intersection.

The structure of HmuY is asymmetric, with maximal dimensions of ∼55×40×35 Å, and it resembles a right hand (Figure 4A). It consists of a roughly globular nucleus reminiscent of a palm, out of which protruding segments mimicking thumb and fingers emerge (Figure 4A, 4B, and 4C). As anticipated by CD spectroscopy (see above and [23]), HmuY is an all β-protein constituted by 15 β-strands. Both the N- and C-terminus are located on the protein surface corresponding to the palm and they point toward the wrist (following the analogy with a hand). They precede and succeed, respectively, two β-strands that participate in a twisted β-sandwich or laterally open β-barrel made up of two antiparallel β-sheets of, respectively, five (sheet I; strands β1+β6+β13−β15; connectivity +4x, −1, −1, −1) and four (sheet II; strands β2−β5; connectivity −1, +2x, +1) strands (Figure 4B and 4C). A large twisted and curled β-ribbon (β7β8) is inserted into the front of the palm mimicking a thumb and two further ribbons are found on the back resembling a pinky (strand β12), a ring finger (β11), a middle finger (β10), and an index finger (β9) (top to bottom in Figure 4C). Sheet I is curled toward sheet II and twisted for ∼95°, while sheet II is arched away from sheet I and twisted for ∼85°. This gives rise to a large hydrophobic cavity at the interface between sheets, which is the core of the protein and contributes to most of the palm. It is created by side chains from strands β1 and β2, the loop connecting β2 with β3 (Lβ2β3), and strands β3−β6 and β13−β15. In addition, hydrophobic residues provided by Lβ6β7, Lβ8β9, and Lβ10β11 close the β-sandwich on the flank bordered by β5 (sheet II) and β6 (sheet I) (Figure 4C). Two smaller hydrophobic cores are observed on the convex side of either sheet by side chains provided by β6, β13−β15, and Lβ6β7, which folds back on top of the sheet (sheet I), and Lβ3β4 *plus* Lβ5β6 (sheet II). All together, these hydrophobic clusters give rise to a molecule, which provides a structural explanation for its high stability and resistance toward denaturation and proteolysis.

### Heme binding in holo-HmuY

Together with the finger-proximal lateral wall of the central hydrophobic core, thumb and fingers give rise to the heme-binding cavity, which is radically different in structure and location within the molecule from those found in any other heme-binding protein described. The cavity occupies a volume of 2,136 Å^3^ and is made up by 37 residues, including 29 hydrophobic or neutral residues and eight charged residues (Figure 4D). A single Fe^3+^-chelating heme b molecule (hemin) is inserted laterally like a wedge into the cavity, curiously with its charged propionate substituents pointing toward the palm and the hydrophobic methyl and vinyl substituents pointing toward the exterior (Figure 4C and 4D). The protein∶heme complex is characterized by an interaction surface of 532 Å^2^, which is 61% of the total accessible surface of the co-factor (868 Å^2^). This value is rather low if compared with other heme-protein complexes [32] but can be explained in terms of the quaternary arrangement (see below). Complex interactions are mainly hydrophobic and entail three hydrogen bonds, one salt bridge, and van-der-Waals interactions between 13 protein residues (Table 1 and Figure 4D). Further noteworthy are a salt-bridge of Arg79 Nη2 and two hydrogen bonds of Tyr80 Oη and Tyr13 Oη with the carboxylate oxygen atoms of the propionate substituent of pyrrol ring a, as well as a hydrogen bond between Thr124 Oγ1 and the propionate of ring d. However, the most relevant contacts are the metallo-organic bonds established between the heme iron and the Nε2 atoms of His134 (2.04 Å away), provided by thumb-strand β8, and His 166 (2.09 Å), from ring-finger-strand β11. These two ligands occupy the apical positions of an octahedral iron co-ordination sphere, whose equatorial ligands are the four porphyrin nitrogen atoms (at 2.03–2.06 Å). Accordingly, ion co-ordination is exerted by six nearly equivalent and equidistant sp^2^-hybridized nitrogen atoms and is thus highly symmetrical, which should redound to a very stable complex. In addition, both protein histidine side chains are in hydrophobic environments: His134 is surrounded by Tyr127, Met129, Met136, and Pro168 (from a vicinal complex; see next chapter) and His166 by Phe156, Phe164, Pro171, and the methyl and vinyl groups from an adjacent complex.

**Table 1:** Protein∶co-factor and protein tetramerization contacts.

HmuY∶heme contacts^a^	Hydrogen bonds and polar interactions and metallo-organic bonds (in Å)			Van-der-Waals interactions performed by protein residues		
	Arg79 Nη2	Hem301 O1A	2.81	Tyr80	Thr124	Tyr127
	Tyr80 Oη	Hem301 O2A	2.59	Met129	Met136	Gly155
	Thr124 Oγ1	Hem301 O1D	2.70	Phe156	Phe164	Ala169
	His134 Nε2	Hem301 FE	2.04	Gly170	Pro171	Tyr173
	His166 Nε2	Hem301 FE	2.09	Lys204		
	Tyr173 Oη	Hem301 O2A	2.60			

Contacts between contiguous monomers (molecules 1-2)^a^^,^ b	Hydrogen bonds and polar interactions (in Å)			Van-der-Waals interactions (in Å)		
	Glu128 Oε1	Lys204 Nζ	2.99/2.97	Met129 Cβ	Hem301 CMA	3.59/3.60
	Pro131 O	Ala169 N	2.86/2.86	Pro131 Cβ	Thr172 Cγ2	3.64/3.64
	Pro131 N	Pro171 O	3.40/3.39	Asp132 C	Ala169 Cβ	3.90/3.88
	Gly133 N	Ala169 O	3.06/3.05	Hem301 CBB	Hem301 CBB	3.55
	Pro168 N	Pro131 O	3.38/3.38			

Contacts between opposite monomers (molecules 1-3)^c^				Van-der-Waals interactions (in Å)		
				Ala169 Cβ	Ala169 Cβ	3.28
				Asp132 Cγ	Asp132 Cγ	4.00
				Hem301 CBB^b^	Hem301 CBB^b^	3.99

a Atom CBB is the terminal vinyl methylene carbon of pyrrole ring b, and atom CMA is the methyl carbon of pyrrole ring a (see Figure 4D for pyrrole naming within the heme protoporphyrin ring). Atoms O1A, O2A, and O1D are carboxylate oxygens of the propionate substituents of rings a and d, respectively.

b All the interactions between molecule 1 and molecule 2 are symmetric. Distances 1-2 and 2-1 are given separated by a slash.

c All the interactions between molecule 1 and molecule 3 are symmetric and made by identical atoms.

### Quaternary arrangement of holo-HmuY

The quaternary structure of holo-HmuY is a cross-like tetramer of complexes, ∼95 Å in diameter, created by the combination of a local and a crystallographic two-fold axis (Figure 4E). This finding fits well with analytical size-exclusion chromatography studies showing that, upon addition of heme, HmuY eluted as a tetramer [23]. The tetramer is made up by interactions between thumb and finger tips and includes β-hairpins β7β8, β9β10, β11β12, and β14β15 of each of the four complexes, which contribute through a surface of 1148–1174 Å^2^ to the oligomer. This is within the range reported for interaction surfaces usually found in protein complexes [33]. All contacts between vicinal complexes (e.g. the magenta and yellow ones in Figure 4E) are symmetric and include eight hydrogen bonds, two salt bridges and four hydrophobic interactions, two hydrophobic protein-heme and one hydrophobic inter-heme contacts (Table 1). Fewer contacts are observed between opposite complexes (e.g. the magenta and cyan ones in Figure 4E); they just include three symmetric hydrophobic interactions established by Ala169, Asp132, and the vinyl substituents of heme pyrrol rings b (Table 1). The overall arrangement entails that the four heme groups are in direct contact with each other in the center of the tetramer. Each co-factor molecule has a buried surface of 636–639 Å^2^, 74% of its total area. The inter-iron distances are 13 Å (vicinal monomers) and 17 Å (opposite monomers), within the range of values observed in electron-transport proteins such as cytochrome c (9–18 Å; [34],[35]), although no function of HmuY in redox biochemistry or electron transport has been postulated. As a result of this packing the four heme groups are buried and thus protected from competing heme-binders.

### Structural aspects of related heme-binding proteins

Structure similarity searches following a variety of algorithms failed to identify significant matches extending beyond selected parts of the central β-sandwich, so we conclude that HmuY conforms to a novel all β-fold. Among structurally characterized heme-binding proteins participating in iron storage and transport are the archetypes hemoglobin and myoglobin, which like related globins and serum albumin, are all-α class proteins [36]–[38]. With respect to functional analogs of HmuY, only the closely-related hemophores HasA and HasAp from *S. marcescens* and *P. aeruginosa*, respectively, have been reported for their structure [18],[28]. They consist of a two layer α/β-sandwich with a meander fold characterized by a twisted antiparallel six-stranded β-sheet with four helices on its concave side. The overall shape of the molecules is reminiscent of a fish, which traps a heme in its mouth. Here, the iron is octahedrally co-ordinated by an apical histidine and a tyrosine. Like HmuY, HasA and HasAp do not undergo major structural rearrangement upon heme binding.

The only other structurally characterized all-β-structure engaged in heme transport and storage is serum hemopexin [32],[39]. It consists of two tandem ∼200-residue fourfold β-propeller domains, which are thick discs consisting of four blades arranged around a central channel. Each blade is made up of a twisted four-stranded antiparallel β-sheet [39]. The functionally relevant oligomerization state is a monomer, and heme binding correlates with major structural rearrangement [32]. In the heme-bound complex, the two hemopexin domains are roughly perpendicular to each other and connected by a partially flexible 20-residue linker (Figure 4F). The heme–binding site resides at the interface between domains, which is covered by a cluster of conserved aromatic residues. As in HmuY, heme binding is exerted by two histidine residues, one provided by the linker, which bind the iron on its two apical positions, and a cluster of basic residues and tyrosines that bind the heme propionate groups [32]. Further as in HmuY, the heme propionate groups are buried in the molecule and the hydrophobic substituents of heme rings b and c likewise point toward the exterior of the molecule. However, beyond these very detailed features and a generally apolar environment of the heme-binding cavity, there is no further structural similarity between holo-hemopexin and holo-HmuY.

### Proposed working mechanism for the Hmu system

**Figure 5:**
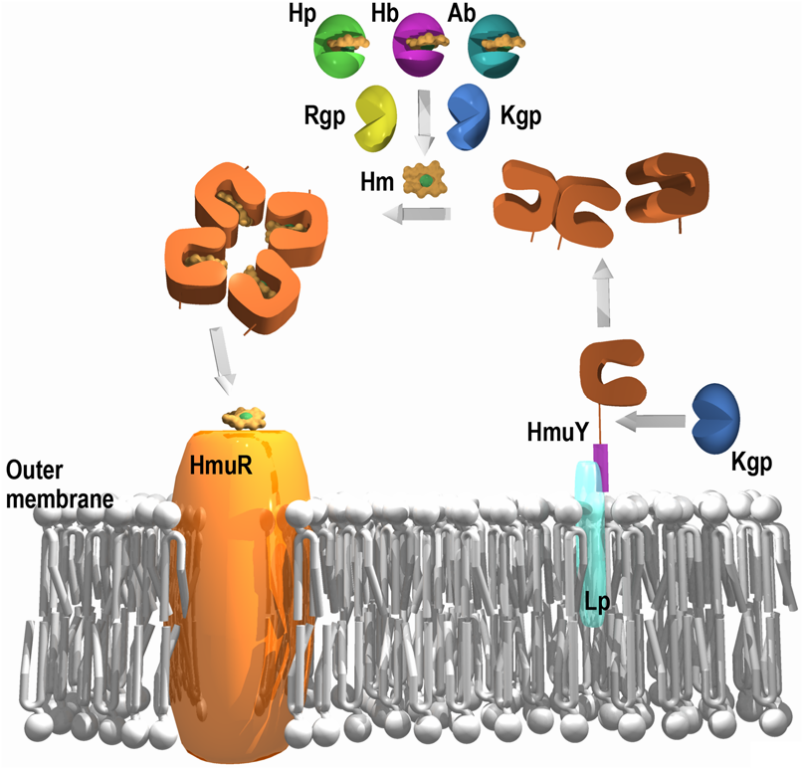
Proposed working mechanism for the extracellular hmu components. HmuY and HmuR are shown with their proposed roles in heme uptake, as well as the other suggested players. Hb stands for hemoglobin; Hp for hemopexin; Ab for serum albumin; Rgp for gingipains R types A and B; Kgp for gingipain K; Lp for lipid; and Hm for heme (represented as in Figure 4A).

Accumulating biochemical and genetic evidence [13], [14], [22]–[25],[40],[41], as well as the present data, suggest the following mechanism for Hmu-mediated heme uptake in *P. gingivalis* and, by extension, in other related bacteria (Figure 5). HmuY is synthesized and exported to the outer membrane, where it would be anchored to a lipid through an attachment site typical for prokaryotic lipoproteins [42]. Location of HmuY at the outer membrane of intact *P. gingivalis* cells and to outer-membrane vesicles has been shown [23],[25],[26]. In addition, the existence of a membrane-attached HmuY species was substantiated by experiments in *Escherichia coli* cells [43]. From the surface, HmuY would be shed by Kgp to enter the inflamed periodontal tissue at the site of infection. This step is backed by N-terminal sequencing of HmuY purified from the culture medium, which revealed that the protein starts with residue Asp26, and that recombinant HmuY comprising five additional upstream residues of the gene-encoded sequence, i.e. starting with Met21-Gly-Lys-Lys-Lys-Asp26, was cleaved at bond Lys25-Asp26 by Kgp *in vitro* (data not shown). Moreover, mRNA encoding HmuY was much more highly expressed than any of the other *hmu*-operon encoded proteins (7–20 times and several orders of magnitude more transcription than HmuR and HmuS-V, respectively [23],[25]). This observation, as well as our results demonstrating high stability against denaturation and proteolysis, is consistent with the idea of a protein that is targeted for secretion as a virulence factor during infection. One of the virulence mechanisms in *P. gingivalis* is biofilm formation, which facilitates the long term survival of the bacterium and induces an inflammatory reaction in the host. A recent report showed that HmuY is found predominantly in the biofilm [44]. Furthermore, it was demonstrated that the *P. gingivalis hmuY*-knockout mutant cannot grow in the presence of human serum as a sole heme source, confirming that this protein is necessary for growth of bacteria under low iron/heme conditions, as found in deep biofilm layers [23],[45].

Host hemoproteins are present in significant concentrations in the gingival crevice [46], but HmuY would not be able to compete with them for heme binding due to its much lower affinity for the co-factor (K_d_∼3 µM, our unpublished data; hemopexin and hemoglobin, K_d_<1 pM, [47],[48]; haptoglobin/hemoglobin complex, K_d_<fM, [49]; serum albumin, K_d_∼10 nM, [50]). However, gingipains, the major proteases produced and secreted by *P. gingivalis*, as well as other secreted proteases, can efficiently cleave hemoglobin, haptoglobin, and hemopexin, thus liberating heme [10], [51]–[54]. In contrast, we have shown here that HmuY is resistant to proteolysis by gingipains. In an alternative or complementary fashion, release of heme from these hemoproteins could occur spontaneously, as this is a low-energy event [14], or through conformational changes induced in the host hemoproteins that would make the co-factor accessible. In any case, HmuY would take up heme, and this would lead to tetramerization under occlusion of the heme binding sites. Tetrameric HmuY would protect heme from host scavengers and shepherd it to HmuR. At this point, heme transfer to the latter encounters two obstacles: the receptor has ten times lower affinity for heme than HmuY (K_d_ = 24 µM; [21],[40],[41]) and the heme groups are inaccessible within the holo-HmuY tetramer. Accordingly, this step would probably entail a rupture of the tetramer triggered by the HmuY-HmuR interaction to expose heme. Similarly, disruption of a tight heme/carrier complex to enable heme uptake by a receptor have been reported for the *S. marcescens* HasA-HasR system [15] and for hemopexin-hemopexin receptor [32]. On the basis of our mutational analysis of HmuY heme ligands [27], an initial step in heme transfer could involve disruption of only one of the two axial histidine ligands as found for HasA [15],[27]. At this point, a Fe(III)-to-Fe(II) transition is conceivable [27]. Once bound by HmuR, heme would be translocated across the outer membrane into the periplasm. In the absence of the HmuR receptor, heme cannot be efficiently transported into the cells, which retards growth of the *hmuR*-knockout mutant [43]. In addition, this mutant becomes more pigmented, indicating that HmuY binds and stores the accumulating excess of heme on the bacterial cell surface as a result of the broken pipeline. Therefore, HmuY, especially in the form associated with the outer membrane, may also store heme and protect the bacterial cell from damage induced by free heme. A phenotype of *P. gingivalis hmuY*- and *hmuY/hmuR*-knockout mutants confirms this hypothesis since both strains are less pigmented than the wild-type [23]. Heme translocation by HmuR putatively occurs under assistance of TonB [11],[14]. The pernicious oxidative potential of free heme would also require the presence of binding proteins to escort it across the periplasm to the cytoplasm [21]. This step might be performed by the other *hmu* operon proteins, which would be required in much less amount than HmuY: HmuS, which displays sequence similarity with cobN/Mg chelatase; HmuT and HmuU, which are similar in sequence to permeases; and HmuV, annotated as an ATP-binding protein engaged in hemin import [25]. Further studies, e.g. of the HmuY/HmuR interaction, are necessary to understand this novel heme transfer mechanism for bacterial survival.

### Conclusions

Pathogenic bacteria have evolved sophisticated mechanisms in response to the changing environment and the host antimicrobial defense systems. The multiprotein system possibly encoded by the *hmu* operon in proteobacteria, bacteroidetes, spirochaetes, and chlorobi, contributes to heme uptake and utilization for bacterial survival and infection. As pathogenic bacteria continue to develop resistance to antibiotics, targeting nutrient uptake systems may offer novel strategies to combat microorganisms such as *P. gingivalis*, a formidable pathogen. In this context, these data on structure and function of the *hmu*-encoded heme-binding protein, HmuY, which may have also a role in the host immune response and in interaction with host cells, may lead to the development of novel therapeutic approaches to pathogen incapacitation. The high stability of HmuY given by its unique structure makes it a suitable candidate for biotechnological and biomedical applications.

## Materials and Methods

### Protein production and purification

*P. gingivalis* apo-HmuY lacking the first 25 residues of the DNA-derived protein sequence (NCBI accession number CAM 31898) and a variant lacking the first 21 residues but containing a C-terminal HSV and His8-tag, were expressed using plasmids pHmuY11 or pDB and *E. coli* ER2566 (New England Biolabs) cells, and purified as previously reported [23],[43]. A protein variant incorporating selenomethionine instead of methionine was prepared using plasmid pHmuY11 and the same cells, which were added to 500 mL of minimal medium lacking methionine and implemented with 25 mg of selenomethionine (Sigma) 30 min before induction [55]. Holo-HmuY was reconstituted from heme and apo-HmuY by incubating 1 equivalent of protein with 1 equivalent of heme (ICN Biomedicals) at room temperature. Excess heme was removed by gel filtration through a PD-10 desalting column (Amersham Pharmacia). To purify HmuY from culture media, *P. gingivalis* cells were cultured anaerobically on blood-agar plates and then in basal medium supplemented with hemin or dipirydyl as described previously [23]. Cultures were centrifuged at 20,000×*g* for 20 min at 4°C, supernatants filtered using membranes with a pore size of 0.22 µm, dialyzed against 50 mM Tris/HCl buffer, 25 mM NaCl, pH 7.6, and concentrated using 10-kDa cut-off membranes (Amicon). Concentrated media were further ultracentrifuged (Beckman) at 100,000×g for 2 h at 4°C and supernatants were used to purify HmuY.

### UV-Vis absorption, circular dichroism (CD), and intrinsic fluorescence spectroscopies

UV-Vis spectra were recorded with an Agilent 8453E UV-Vis spectrophotometer (Agilent Technologies). Far-UV CD spectroscopy (205–255 nm) was carried out using a Jasco J-810 spectropolarimeter and 10-mm-path-length cuvettes. For thermal denaturation experiments, CD spectra were recorded from 210 to 250 nm. The CD signals at 225 nm were monitored as a function of temperature from 20 to 80°C. Protein samples were examined in 20 mM sodium phosphate, 20 mM NaCl, pH 7.4 or 20 mM sodium phosphate, 1 M GdnHCl, pH 6.5.

GdnHCl unfolding experiments were performed according to standard protocols [56]. A stock of 6 M GdnHCl (MP Biochemicals) was used to prepare solutions in 20 mM sodium phosphate, 20 mM NaCl, pH 7.4 and variable GdnHCl concentrations (0 to 6 M). Subsequently, concentrations of GdnHCl solutions were determined through measurement of their refractive index at 25°C using a Zeiss refractometer. Apo- and holo-HmuY (protein∶co-factor ratio 1∶1) were added to each sample at 2 µM final concentration and incubated at 25°C for 18 h. GdnHCl-induced chemical denaturation was monitored at 20°C by CD using a Jasco J-810 spectropolarimeter and by intrinsic tryptophan fluorescence using a Jasco FP-750 spectrofluorometer. Samples were excited at 295 nm for fluorescence measurements and the emission spectra from 300 to 700 nm were recorded (slit width 5 nm). CD and fluorescence data were transformed to yield the relative fraction of unfolded protein and to determine the free energy of denaturation.

### Susceptibility to proteolysis

HmuY was subjected to proteolysis by trypsin, Kgp, RgpA, and RgpB. For tryptic digestion, two reactions with HmuY in 100 mM Tris/HCl, 20 mM CaCl_2_, pH 8.0 at 1∶50 protease∶substrate molar ratio were conducted in the presence (1∶1 molar ratio) and absence of heme. Fresh portions of trypsin were added every 1 h during the first 12 h, and then every 12 h. For assays with gingipains, proteases were pre-incubated in 200 mM HEPES, 10 mM cysteine, pH 7.6 for 15 min at 37°C prior to addition of HmuY purified from *E. coli* cells or *P. gingivalis* culture medium (1∶20 protease∶substrate molar ratio) and further incubation for 1 or 20 h at 37°C. Aliquots were taken from the reaction mixtures at given time points, and the reaction was inhibited by addition of a protease-inhibitor cocktail (Roche) and through boiling in SDS-PAGE sample buffer. Control reactions were performed in the absence of protease. HmuY samples previously subjected to thermal denaturation (95°C, 10 min) were also assayed for proteolytic susceptibility. All samples were examined by 15%-Tris/glycine SDS-PAGE and Coomassie Brilliant Blue G-250 staining.

### Crystallization and diffraction data collection

Crystallization assays were performed following the sitting-drop vapor diffusion method. Reservoir solutions were prepared by a Tecan robot and 200-nL crystallization drops were dispensed on 96×2-well MRC plates (Wilden/Innovadyne) by a Cartesian nanodrop robot (Genomic Solutions) at the joint IBMB-CSIC/IRB/Barcelona Science Park High-Throughput Crystallography Platform (PAC). Best crystals as thin reddish prisms appeared in a Bruker steady-temperature crystal farm at 20°C using protein solution (26 mg/mL in 5 mM Tris/HCl, pH 8.0) and 2.4 M (NH_4_)_2_SO_4_, 0.1 M MES, pH 6.0 as reservoir solution and D(+)-glucose monohydrate as additive (relative volumetric ratios 1∶1∶0.35). These conditions were successfully scaled up to the microliter range with Cryschem crystallization dishes (Hampton Research). Crystals of selenomethionine-derivatized protein were obtained under similar conditions. Cryoprotection of protein crystals for diffraction data collection was achieved through harvesting with 3.0 M (NH_4_)_2_SO_4_, 0.1 M MES, pH 6.0 and subsequent stepwise replacement of the mother liquor with 3.0 M (NH_4_)_2_SO_4_, 25% glycerol, 0.1 M MES, pH 6.0. Complete diffraction datasets were collected at 100 K from a single flash-cryo-cooled (Oxford Cryosystems) native crystal (at λ = 1.0000 Å) and from a derivatized crystal (at λ = 0.9792 Å; absorption peak for selenium determined through a fluorescence scan performed with a Si-drift chamber detector (Rontec)) on an ADSC Q315R CCD detector at beam line ID23-1 of the European Synchrotron Radiation Facility (ESRF, Grenoble, France) within the Block Allocation Group “BAG Barcelona”. Crystals were tetragonal and harbored one dimer per asymmetric unit (V_M_ = 2.5 Å^3^/Da; 58% solvent contents). Diffraction data were integrated, scaled, merged, and reduced with programs XDS [57] and SCALA within the CCP4 suite [58] (see Table 2).

**Table 2:** Crystallographic data.

Dataset	Native	Seleno-Methionine Derivative
Space group/cell constants (a and c, in Å)	P4_2_2_1_2/93.74; 113.73	P4_2_2_1_2/93.64; 113.77
Number of measurements/unique reflections	613,987/47,568	320,816/29,379
Resolution range (Å) (outermost shell)	48.6–1.80 (1.90–1.80)	48.6–2.11 (2.22–2.11)
Completeness (%)	100.0 (99.9)	98.4 (89.2)
R_r.i.m._ ( = R_meas_)/R_p.i.m_ a ^,^ b ^,^ c	0.101(0.696)/0.028(0.190)	0.081(0.224)/0.027(0.089)
Average intensity over st. dev. (<[<I>/σ(<I>)]>)	23.0 (4.2)	23.9 (7.3)
B-factor (Wilson) (Å^2^)/average multiplicity	17.6/12.9 (13.1)	21.0/10.9 (5.9)
Heavy-atom sites used for phasing/*fom* d		9/0.68, 0.84
Resolution range used for refinement (Å)	48.6–1.80	
Number of reflections used (test set)	46,847 (720)	
Crystallographic R_factor_ (free R_factor_)^e^	0.160 (0.187)	
No. of protein atoms^f^/solvent molecules/ligands/ions	2,870/432/2 heme (with Fe^3+^); 6 glycerols/5 SO_4_^2−^	
*Rmsd* from target values		
bonds (Å)/angles (°)	0.012/1.31	
bonded B-factors (main chain/side chain) (Å^2^)	0.70/2.19	
Average B-factors for protein atoms (Å^2^)	14.1	
Main-chain conformational angle analysis for residues in favored regions/outliers/all residues	349/0/360	

a Friedel mates were treated as independent reflections in the derivative dataset.

b Values in parentheses refer to the outermost resolution shell.

c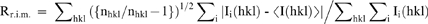 and 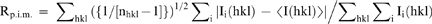.

d Mean figure or merit computed for data to 1.8 Å before and after density modification with program DM within CCP4.

e Crystallographic R_factor_ = Σ_hkl_ ||F_obs_| − k |F_calc_||/Σ_hkl_ |F_obs_|; free R_factor_, same for a test set of reflections not used during refinement.

f Including atoms in alternate conformation.

### Structure solution and refinement

The structure was solved by SIRAS by using native and selenomethionine-derivative diffraction data and programs SHELXD/E under phase extension to 1.8 Å [59]. The phases obtained were subjected to a density modification step with program DM within CCP4 and an electron density map was computed. Subsequently, manual model building on a Silicon-Graphics workstation using program TURBO-Frodo alternated with crystallographic refinement with REFMAC5 within the CCP4 suite until completion of the model (see Table 2). This model contained protein residues Glu35 to Lys216 *plus* a heme co-factor for each of the two protein chains within the crystal asymmetric unit (molecules A and B). The ten N-terminal residues of the protein were flexible and were not traced. Both molecules were equivalent in practice (*rms* deviation for all atoms equaled 0.57 Å), so discussion of the structure considered only molecule A unless otherwise stated.

### Miscellaneous

Figures were prepared with programs SETOR [60] and Carrara 4. Structural similarity searches were performed with programs DALI (http://ekhidna.biocenter.helsinki.fi/ dali_server), SSM (http://www.ebi.ac.uk/msd-srv/ssm), VAST (http://www.ncbi.nlm.nih.gov/Structure/VAST/vast.shtml), and the CATHEDRAL server (http://www.cathdb.info/). Cavity volumes were computed with program PDBsum (http://www.ebi.ac.uk/pdbsum). The final co-ordinates of holo-HmuY have been deposited with the PDB at www.pdb.org (access code 3H8T). Inter- and intra-molecular close contacts (<4 Å) and contact surfaces (with a rolling sphere of 1.4 Å) were determined with program CNS [61]. The value of interaction surfaces was estimated taking the half of the difference between the sum of the individual molecular surfaces and the total complex surface.
